# Grid Map Fusion Method for Multiple Mobile Robots in Large-Scale Packaging and Printing Workshops

**DOI:** 10.3390/s26195993

**Published:** 2026-09-22

**Authors:** Xingmei Wei, Haitao Hao, Jiahao Wang, Jian Li, Kui He

**Affiliations:** 1 School of Mechatronics Engineering, Henan University of Science and Technology, Luoyang 471000, China; 2 High-End Bearing Collaborative Innovation Center of Henan Province, Luoyang 471000, China

**Keywords:** grid map fusion, multi-resolution cascading, optimal transport, local topological constraints, multi-robot cooperative mapping

## Abstract

To address the challenges in large-scale packaging and printing workshops, where the dense arrangement of electromechanical equipment leads to large-scale scenes and prominent repetitive structures, and where frequent dynamic disturbances impair the accuracy and efficiency of cooperative mapping by multiple mobile robots, this paper proposes a grid map fusion method that integrates multi-resolution cascading with optimal transport. The method constructs a coarse-to-fine pyramid hierarchy: at the coarse-resolution level, an initial estimate of the global alignment trend is obtained, and a cross-layer prior transfer mechanism maps the prior information to the finer level, with log-domain Sinkhorn iterations ensuring numerical stability in fine matching; at each resolution level, Oriented FAST and Rotated BRIEF (ORB) features are extracted, and a hybrid cost matrix is formulated by combining appearance information with local topological signatures, while virtual nodes are introduced to improve robustness against missing correspondences and dynamic outliers; high-confidence correspondence candidates are selected based on bidirectional normalized confidence and the maximum consensus criterion, and the Random Sample Consensus (RANSAC) algorithm is employed to estimate the two-dimensional rigid-body transformation; finally, a globally consistent grid map is generated through standard log-odds probability fusion. Experiments on a self-constructed packaging and printing workshop dataset demonstrate that the proposed method maintains registration accuracy while effectively reducing the computational burden of large-scale map fusion, thus providing a feasible technical pathway for global environment modeling and dynamic task scheduling in multi-robot cooperative operations.

## 1. Introduction

Multi-robot collaborative mapping provides an essential foundation for environmental perception, autonomous navigation, and flexible logistics scheduling in smart factories. By sharing local observations, pose estimates, and map information, multi-robot systems can extend environmental coverage while improving mapping efficiency and robustness in large-scale environments [1]. With the rapid development of vision, LiDAR, and multi-sensor technologies, single-robot simultaneous localization and mapping (SLAM) has evolved into a relatively mature technical framework. Methods based on local visual features have been extensively investigated [2], while systems such as ORB-SLAM3 have further advanced visual, visual–inertial, and multi-map SLAM capabilities [3]. Building on these developments, multi-robot collaborative SLAM has attracted increasing research attention, and recent reviews have systematically summarized key issues including map sharing, data association, relative localization, and global consistency [4]. In recent years, substantial progress has been made in distributed multi-robot SLAM [5,6], inter-robot relative localization [7], collaborative active SLAM [8], LiDAR-based collaborative mapping [9], and cooperative exploration in unknown environments [10].

In large-scale packaging and printing workshops, mobile robots are required to operate across multiple functional areas, including raw-paper storage zones, printing-unit areas, finished-product palletizing zones, and logistics corridors, while integrating local maps constructed at different times and along different trajectories into a unified global map. Compared with conventional indoor environments, such industrial settings are characterized by densely deployed equipment, highly repetitive structures, and frequent dynamic disturbances. Previous studies on automated guided vehicles (AGVs) have shown that personnel, transport vehicles, and temporary obstacles can substantially increase the difficulty of robot localization and navigation [11], while dynamic obstacle perception and autonomous collision avoidance are also critical to improving the autonomous operation of industrial mobile robots [12]. Research on data fusion for multi-robot collaborative SLAM has further demonstrated that integrating observations from multiple sources can improve both the completeness of environmental representations and overall map consistency [13]. For large-scale environments, fast registration and fusion of occupancy grid maps have also been investigated [14]. Nevertheless, in packaging and printing workshops, regularly arranged printing units, conveyor lines, shelving systems, and pallets tend to produce numerous geometrically similar boundaries. At the same time, forklifts, personnel, and temporarily stored objects can introduce occlusions, missing correspondences, and spurious matches, thereby further increasing the difficulty of reliable map fusion.

Among existing approaches to multi-robot occupancy grid map fusion, feature-based matching methods can establish spatial correspondences between local maps without requiring highly accurate initial relative poses. Sunil et al. proposed a feature-based occupancy map-merging method for collaborative SLAM, in which local feature associations are used to register maps generated by multiple robots [15]. Wang et al. improved submap-merging consistency by jointly optimizing the global occupancy map and the coordinate frames of local submaps [16]. Chao et al. provided a systematic review of map-matching algorithms and summarized the major strategies used for establishing reliable correspondences [17]. In addition, Tao et al. investigated the fusion of occupancy grid maps with different resolutions [18], Andersone proposed an asymmetric fusion method for heterogeneous occupancy grid maps [19], and Sisojevs et al. studied heterogeneous map fusion from the perspective of occupancy grid histograms [20]. These studies provide an important foundation for integrating maps acquired from different sources and at different spatial resolutions. However, in industrial environments characterized simultaneously by large spatial scales, highly repetitive structures, and frequent dynamic disturbances, further improvements are still required in terms of matching accuracy, robustness, and computational efficiency.

To enhance the reliability of feature association in complex environments, Sarlin et al. proposed SuperGlue, which jointly models local features and their contextual relationships using graph neural networks, thereby improving matching performance in challenging scenes [21]. Lajoie et al. proposed DOOR-SLAM, which employs distributed outlier detection to improve the robustness of multi-robot SLAM against erroneous constraints [22]. Studies on occupancy grid map fusion without prior knowledge of the initial relative poses have further demonstrated that reliable local matching and effective suppression of incorrect correspondences are crucial for maintaining global map consistency [23]. Nevertheless, directly performing exhaustive matching on large-scale, high-resolution maps still faces the following challenges:

1. As the map size and the number of feature points increase, the candidate search space expands substantially, leading to higher computational costs for feature matching [14,15,16,17];

2. Repetitive structures, such as equipment boundaries, parallel corridors, and regularly arranged stacks, tend to generate locally similar features, thereby increasing matching ambiguity [15,17,20,21];

3. Personnel, forklifts, and temporary obstacles can introduce occlusions and spurious correspondences, which may adversely affect relative pose estimation and global map consistency [11,12,22,23];

4. Although multi-resolution processing can reduce the computational burden of feature matching, directly rescaling occupancy probability maps may blur the distinction between the representation used for matching and that used for probabilistic map fusion [18,19,20].

To address these challenges, this study proposes a multi-resolution cascaded optimal-transport method for occupancy grid map fusion in large-scale packaging and printing workshops. The proposed method distinguishes between the matching representation and the fusion representation of local maps. While preserving the original occupancy probabilities or log-odds values for probabilistic fusion, a coarse-to-fine multi-resolution pyramid is constructed specifically for map matching. The coarse-resolution level is used to reduce the search space and estimate the global alignment tendency, after which a cross-layer prior transfer mechanism is employed to guide fine-resolution matching. ORB-based appearance descriptors and local topological signatures are jointly incorporated into a hybrid matching cost, while virtual nodes are introduced to accommodate missing correspondences and anomalous matches. On this basis, an entropy-regularized optimal transport model with cross-layer prior constraints is formulated, and log-domain Sinkhorn iterations are adopted to obtain numerically stable soft correspondences. High-confidence correspondences are subsequently extracted and used to estimate the two-dimensional rigid-body transformation via RANSAC. Finally, the aligned local maps are fused using a standard log-odds formulation. The overall workflow of the proposed method is illustrated in Figure 1.

This study is specifically oriented toward large-scale packaging and printing workshops as a representative industrial scenario. A unified framework is developed to integrate multi-resolution candidate reduction, local structure-constrained matching, partial correspondence estimation, and probabilistic map fusion, with the aim of balancing computational efficiency, registration accuracy, and practical applicability.

## 2. Cascaded Optimal Transport Solving Framework

### 2.1. Multi-Resolution Representation Construction

To reduce the high computational cost incurred by direct matching of large-scale local grid maps, this paper constructs a multi-resolution representation for each local map, with the model illustrated in Figure 2. To prevent multi-scale processing from obscuring the probabilistic semantics of the grid map, this paper distinguishes between two representations: the fusion representation is retained as the original log-odds map, while the matching representation is a normalized grayscale image.

Let the highest resolution level be l=0 and the lowest be l=L, and denote the matching representation at level *l* as *I*(*l*). The matching representation at level *l* + 1 is then obtained from level *l* via Gaussian smoothing and downsampling:

(1) $$I^{\left(l+1\right)}=D(G_{\sigma_{l}}\times I^{\left(l\right)}),l=0,1,\ldots ,L-1$$

In this expression, $G_{\sigma_{l}}$ is the Gaussian kernel with scale parameter $\sigma_{l}$, and D denotes the downsampling operator. A schematic diagram of Gaussian downsampling is shown in Figure 3. As the pyramid level deepens, the map resolution gradually decreases, and the number of feature points as well as the number of candidate matches is reduced synchronously, while the overall structural outline of the scene remains preserved.

In large-scale packaging and printing workshops, the coarse resolution level is primarily used to rapidly capture the orientation of main aisles, the layout of equipment, and the large-scale overlap trend, thereby achieving preliminary alignment across the entire scene; the fine resolution level is then employed to recover equipment edges, stack contours, and local obstacle details, thus supporting subsequent fine matching. Compared with performing full matching directly at the original resolution, the multi-resolution representation can effectively compress the search space and provide more reliable initial guidance for the fine-level optimization.

### 2.2. Cross-Layer Prior Transfer and Warm Start

In the conventional single-level optimal transport solving process, the matchings at different levels are mutually independent, making it difficult to effectively exploit the global structural information already obtained at the coarse resolution level. To this end, this paper designs a cross-layer prior transfer and warm-start mechanism that maps the optimal transport result from the coarse resolution level to serve as an initialization prior for the fine-level solution, thereby reducing the fine-level search space and enhancing optimization stability.

Denote the coarse-level source feature point set as $P_{A}^{\left(l+1\right)}=\{p_{i}\}$ and the target feature point set as $P_{B}^{\left(l+1\right)}=\{q_{j}\}$; denote the fine-level source feature point set as $P_{A}^{\left(l\right)}=\{p_{u}^{\prime }\}$ and the target feature point set as $P_{B}^{\left(l\right)}=\{q_{v}^{\prime }\}$. To establish cross-scale correspondences, the spatial coordinates of the feature points at each level are uniformly transformed into the original map coordinate system. Let $x_{i}^{\left(l+1\right)}$ and $x_{u}^{\left(l\right)}$ be the coordinates of the coarse-level and fine-level source feature points, respectively, and $y_{j}^{\left(l+1\right)}$ and $y_{v}^{\left(l\right)}$ be the coordinates of the coarse-level and fine-level target feature points, respectively.

Define the spatial similarity function $\kappa \left(d\right)=exp\left(-d^{2}/\left(2\sigma_{s}^{2}\right)\right)$, where $\sigma_{s}$ is taken as twice the physical size of the coarse-level pixel. For any coarse-level matched pair (i,j), its assignment coefficient to a fine-level candidate pair (u,v) is defined as:

(2) $$\rho_{uv|ij}=\frac{\kappa \left(x_{u}^{\left(l\right)}-x_{i}^{\left(l+1\right)}\right)\cdot \kappa \left(y_{v}^{\left(l\right)}-y_{j}^{\left(l+1\right)}\right)}{\sum_{u^{\prime },v^{\prime }}\kappa \left(x_{u^{\prime }}^{\left(l\right)}-x_{i}^{\left(l+1\right)}\right)\cdot \kappa \left(y_{v^{\prime }}^{\left(l\right)}-y_{j}^{\left(l+1\right)}\right)}$$

The assignment coefficient satisfies $\sum_{u,v}\rho_{uv|ij}=1$, which means that each coarse-level matching weight is distributed to all candidate pairs in the corresponding fine-level region according to their spatial proximity relationships. Based on this, the prior support matrix $P^{\left(l\right)}$ for fine-level candidates is obtained by mapping from the coarse-level coupling matrix $\Gamma^{\left(l+1\right)}$:

(3) $$P_{uv}^{\left(l\right)}=\sum_{i,j}\rho_{uv|ij}\Gamma_{ij}^{\left(l+1\right)}$$

Since the elements of the coarse-level coupling matrix $\Gamma^{\left(l+1\right)}$ sum to 1 and the assignment coefficients satisfy normalization, it follows that the elements of $P^{\left(l\right)}$ also sum to 1, which can thus be regarded as a prior probability distribution for the fine-level matching. This treatment prevents the unmatched mass associated with virtual nodes from being erroneously propagated to the subsequent refinement level.

Merely incorporating $P^{\left(l\right)}$ into the objective function serves as a cross-level prior constraint and, by itself, does not constitute a warm start in the numerical sense. To ensure consistency between the algorithmic implementation and the notion of a “warm start,” the dual variables obtained after convergence at the coarse level are further propagated to the finer level. Let $N_{A}(u)$ and $N_{B}(v)$ denote the coarse-level neighborhoods corresponding to the fine-level nodes uu and vv, respectively. Let $\omega_{ui}^{A}$ and $\omega_{vj}^{B}$ be the normalized interpolation weights computed according to the spatial similarity function κ(⋅). The fine-level dual variables are then initialized as:

(4) $$f_{u}^{\left(0,l\right)}=\underset{i\in N_{A}(u)}{\sum }\omega_{ui}^{A}f_{i}^{\left(*,l+1\right)},g_{v}^{\left(0,l\right)}=\underset{j\in N_{B}(v)}{\sum }\omega_{vj}^{B}g_{j}^{\left(*,l+1\right)}$$

Here, $f_{i}^{\left(*,l+1\right)}$ and $g_{j}^{\left(*,l+1\right)}$ denote the converged dual variables at the coarse level. Because the optimal transport dual variables are non-unique under additive shifts, the coarse-level dual variables are normalized to have zero mean before inter-level interpolation. The corresponding shift is compensated for in the dual variable on the opposite side so that the coupling matrix remains unchanged. For virtual nodes at the fine level, their dual variables directly inherit the converged values of the corresponding virtual nodes at the coarse level.

For the coarsest resolution level *L*, where no information from a preceding level is available, the dual variables are initialized as $f^{\left(0,L\right)}=0,g^{\left(0,L\right)}=0$.

And the cross-level prior weight is set to $\beta_{L}=0$. For all remaining levels, the interpolation results given in Equation (4) are used to initialize the dual variables. In this manner, the coarse-level solution is used not only to construct the matching prior for the finer level but also to directly initialize the fine-level iterative variables, thereby establishing a complete coarse-to-fine warm-start scheme.

For the long corridors, parallel equipment boundaries, and repetitive contour structures that widely exist in large-scale packaging and printing workshops, the overall spatial layout information provided by the coarse-resolution level matching can effectively guide the fine-level optimization, converting the fine-level search from a global blind search into a locally constrained search, thereby improving the matching efficiency and stability in complex industrial environments.

### 2.3. Regularized Cascaded Sinkhorn Solver

In the multi-resolution cascaded solving process, the requirements on the properties of the optimal transport solution differ between the coarse and fine resolution levels. The coarse level emphasizes preserving global search capability and solution smoothness, so as to rapidly establish candidate overlapping regions and the dominant alignment trend; The fine level focuses on improving local discriminability and enhancing the concentration of transport weights, thereby obtaining high-quality correspondences suitable for geometric estimation. In this process, the coarsest level *L* has no available cross-layer prior, so the prior weight is forced to zero, i.e., $\beta_{l}=0$, and this level reduces to the standard entropy-regularized optimal transport problem. For the remaining levels (*l* < *L*), the prior matrix $P^{\left(l\right)}$ propagated from the coarse level is incorporated into the objective function. Let ${\overset{―}{C}}^{\left(l\right)}$ denote the augmented cost matrix including virtual nodes, ${\overset{\sim }{\mu }}^{\left(l\right)}$ and ${\overset{\sim }{\nu }}^{\left(l\right)}$ denote the augmented marginal distributions, and $\Gamma_{rr}^{\left(l\right)}$ denote the coupling submatrix between the real nodes at the current level. The optimal transport model at level *l* is then formulated as follows:

(5) $$\underset{\Gamma^{\left(l\right)}\in U({\overset{\sim }{\mu }}^{\left(l\right)},{\overset{\sim }{\nu }}^{\left(l\right)})}{min}[\langle {\overset{―}{C}}^{\left(l\right)},\Gamma^{\left(l\right)}\rangle -\varepsilon_{l}H(\Gamma^{\left(l\right)})-\beta_{l}\langle log(P^{\left(l\right)}+\delta ),\Gamma_{rr}^{\left(l\right)}\rangle ]$$

In Equation (5), $U({\overset{\sim }{\mu }}^{\left(l\right)},{\overset{\sim }{\nu }}^{\left(l\right)})$ denotes the set of couplings satisfying the marginal constraints, H(Γ) is the entropy term, $\varepsilon_{l}$ is the entropy regularization coefficient, and $\beta_{l}$ denotes the cross-level prior weight. A small constant *δ* = 10^−6^ is introduced to prevent numerical instabilities in logarithmic computations.

The cross-level prior term in Equation (5) is applied only to the transport block between real nodes and does not act on the transport relationships between real and virtual nodes or between the two virtual nodes. This restriction prevents unmatched mass at the coarse level from imposing erroneous constraints on the matching of real nodes at the finer level.

To facilitate the use of the standard Sinkhorn iteration, the modified cost matrix is defined as follows:

(6) $${\overset{\sim }{C}}_{uv}^{\left(l\right)}=\{\begin{array}{cc} {\overset{―}{C}}_{uv}^{\left(l\right)}-\beta_{l}log(P_{uv}^{\left(l\right)}+\delta ), & u\leq N_{s},\;v\leq N_{t}\\ {\overset{―}{C}}_{uv}^{\left(l\right)}, & \mathrm{otherwise} \end{array}$$

Considering that the fine-level cost matrix typically exhibits a relatively large dynamic range and is associated with a small regularization coefficient, directly performing Sinkhorn iterations in the primal domain may lead to exponential underflow and numerical instability. Therefore, the Sinkhorn algorithm is implemented in the log domain to improve numerical stability. The dual variables are updated according to:

(7) $$f^{\left(t+1\right)}=\varepsilon_{l}[log{\overset{\sim }{\mu }}^{\left(l\right)}-LSE(\frac{g_{j}^{\left(t\right)}-{\overset{\sim }{C}}_{ij}^{\left(l\right)}}{\varepsilon_{l}})]$$

(8) $$g^{\left(t+1\right)}=\varepsilon_{l}[log{\overset{\sim }{\nu }}^{\left(l\right)}-LSE(\frac{f_{i}^{\left(t+1\right)}-{\overset{\sim }{C}}_{ij}^{\left(l\right)}}{\varepsilon_{l}})]$$

In the above expression, LSE denotes the log-sum-exp operator. At the coarsest level, the dual variables are initialized to zero, whereas at all remaining levels they are initialized using the cross-level warm-start results given by Equation (4). Upon convergence of the iterative procedure, the final coupling matrix is reconstructed as follows:

(9) $$\Gamma^{\left(l\right)}=exp(\frac{f^{\left(t\right)}⊕g^{\left(t\right)}-{\overset{\sim }{C}}^{\left(l\right)}}{\varepsilon_{l}})$$

To balance the global search capability at the coarse level and the fine discriminability at the fine level, this paper adopts a dynamic regularization scheduling strategy, such that the regularization strength gradually decreases as the resolution level descends:

(10) $$\varepsilon_{L}>\varepsilon_{L-1}>\cdots >\varepsilon_{0}$$

A larger regularization parameter at the coarse resolution level helps maintain the smoothness of the coupling matrix and prevents the matching process from prematurely falling into local optima caused by repetitive structures; a smaller regularization parameter at the fine resolution level facilitates a more concentrated matching distribution, thereby improving the sparsity and discriminability of the correspondences.

After obtaining the coupling matrix at the finest resolution level, this paper does not adopt the direct assignment approach via row maximum. Instead, the coupling matrix is regarded as a soft matching result, and a set of high-confidence candidate correspondences is extracted from it based on the bidirectional normalized confidence and the bidirectional maximum consensus criterion defined in Section 2.4. This set then serves as the input for the subsequent RANSAC geometric consistency estimation. Through the synergistic effect of multi-resolution cascading, cross-layer prior transfer, and dynamic regularization scheduling, the proposed method rapidly establishes the global alignment trend at the coarse level and achieves fine registration at the fine level using high-confidence sparse correspondences, thereby balancing the solution efficiency and matching accuracy for large-scale workshop map fusion.

### 2.4. High-Confidence Match Extraction

Among the optimal transport results at each level, the coupling matrix $\Gamma^{\left(0\right)}$ at the finest level is capable of describing the soft matching relationships between the feature points of the source map and the target map in a relatively detailed manner. To ensure the stability of the subsequent rigid-body transformation estimation, this paper does not directly adopt all soft matches; instead, high-confidence candidate correspondences are further extracted.

Let $\Gamma_{ij}^{\left(0\right)}$ denote the coupling weight between the source feature point $p_{i}^{A}$ and the target feature point $q_{j}^{B}$. Since the output of entropy-regularized optimal transport is typically a soft assignment, directly applying a fixed threshold to $\Gamma_{ij}^{\left(0\right)}$ is susceptible to the influence of the number of feature points, the marginal distributions, and the regularization strength. To enhance the robustness of candidate selection, this paper defines the bidirectional normalized confidence as:

(11) $$r_{ij}=min\left(\frac{\Gamma_{ij}^{\left(0\right)}}{\sum_{j}\Gamma_{ij}^{\left(0\right)}},\frac{\Gamma_{ij}^{\left(0\right)}}{\sum_{i}\Gamma_{ij}^{\left(0\right)}}\right)$$

Based on this, a set of high-confidence candidates is constructed as follows:

(12) $$S_{cand}=\left\{\left(p_{i}^{A},q_{j}^{B}\right)|r_{ij}\geq \eta \right\}$$

In this expression, *η* is the confidence threshold. To further suppress ambiguities caused by one-to-many or many-to-one matches, the bidirectional maximum consensus principle is applied to filter the candidates, yielding the final set of high-confidence matches:

(13) $$S_{in}=\left\{\left(p_{i}^{A},q_{j}^{B}\right)\in S_{cand}|j=arg{max}_{k}r_{ik},i=arg{max}_{k}r_{kj}\right\}$$

After the above processing, unstable soft matches caused by repetitive structures, partial occlusions, and virtual nodes can be effectively eliminated, thereby providing more reliable input for the subsequent geometric consistency estimation.

## 3. Matching Cost Modeling

### 3.1. ORB Features and Appearance Descriptors

At resolution level *l*, let the source local map and the target local map be denoted as $M_{A}^{\left(l\right)}$ and $M_{B}^{\left(l\right)}$, respectively. Oriented FAST and Rotated BRIEF (ORB) feature points and their descriptors are extracted from their corresponding matching representations, yielding the feature point sets of the two maps as follows:

(14) $$P_{A}^{\left(l\right)}=\{p_{1}^{A},p_{2}^{A},\ldots ,p_{n}^{A}\},P_{B}^{\left(l\right)}=\{q_{1}^{B},q_{2}^{B},\ldots ,q_{m}^{B}\}$$

In the above expression, the binary descriptors of the feature points $p_{i}^{A}$ and $q_{j}^{B}$ are denoted as $d_{i}^{A}$ and $d_{j}^{B}$, respectively.

ORB features offer advantages such as high extraction efficiency, compact descriptors, and suitability for large-scale real-time matching, making them well-suited for multi-robot local map fusion tasks. However, in large-scale packaging and printing workshops, pallet boundaries, unit contours, conveyor guardrails, and long corridor structures exhibit strong repetitiveness, and relying solely on local appearance description is prone to generating false correspondences. For this reason, this paper uses the ORB descriptor as the basic appearance information and combines it with local topological information to jointly construct the matching cost.

For any candidate point pair $\left(p_{i}^{A},q_{j}^{B}\right)$, the local appearance difference is expressed by the normalized Hamming distance as:

(15) $$D_{\mathrm{desc}}\left(p_{i}^{A},q_{j}^{B}\right)=\frac{1}{d_{max}}\mathrm{Ham}\left(d_{i}^{A},d_{j}^{B}\right)$$

In this expression, Ham denotes the Hamming distance, and $d_{max}$ is the length of the descriptor.

### 3.2. Median k-Nearest Neighbor Graph and Topological Signatures

To enhance the discriminability of the matching process for repetitive structures and local ambiguities, this paper constructs a median k-nearest neighbor graph on the feature point set at each level, with the median-based *k*-nearest-neighbor graph construction and edge-pruning procedure illustrated in Figure 4. For each feature point, its k nearest neighbors are searched, and the median of the lengths of the neighboring edges is used as the local scale reference; only edges with lengths not exceeding this median are retained, thereby suppressing the interference of anomalously long edges. In this paper, the value of *k* is set to 10.

On this basis, a local topological signature $s_{i}$ is constructed for each feature point. Considering that unregistered local maps typically exhibit an overall rotational difference, directly using absolute directional information in the global coordinate system would compromise the stability of the topological description. To this end, this paper constructs the topological descriptor using relative angle statistics normalized by the local dominant direction:

(16) $$s_{i}=[deg(p_{i}),\mu_{i},\sigma_{i},h_{i}]$$

In this expression, $deg(p_{i})$ denotes the node degree, $\mu_{i}$ and $\sigma_{i}$ represent the mean and standard deviation of the neighbor edge lengths, respectively, and $h_{i}$ denotes the relative angle histogram constructed based on the local dominant direction. Since $h_{i}$ describes a normalized relative angle distribution, it exhibits better rotational robustness compared with absolute directional features.

For any candidate point pair $\left(p_{i}^{A},q_{j}^{B}\right)$, the topological difference is defined as:

(17) $$D_{\mathrm{topo}}(p_{i}^{A},q_{j}^{B})={∥s_{i}^{A}-s_{j}^{B}∥}_{1}$$

This requirement means that candidate correspondences should be similar not only in local appearance but also consistent in neighborhood structure. For the regular equipment boundaries, parallel contours, and repetitive stacking structures commonly encountered in packaging and printing workshops, this topological constraint can effectively distinguish feature points that are locally similar in appearance but differ in their spatial structural positions, thereby enhancing matching discriminability.

### 3.3. Virtual Nodes and Hybrid Cost Matrix

In large-scale packaging and printing workshops, the multi-robot mapping process is susceptible to viewpoint differences, dynamic obstacles, partial occlusions, and temporary stacked items, which lead to an unequal number of feature points in the two local maps, and not all real feature points possess reliable correspondences. If a strict one-to-one matching model is adopted, outliers may be forcibly assigned to incorrect correspondences. To enhance the model’s adaptability to missing correspondences and outliers, this paper introduces a virtual node into the source feature point set and the target feature point set, respectively, thereby constructing an augmented feature set:

(18) $${\overset{\sim }{P}}_{A}^{\left(l\right)}=P_{A}^{\left(l\right)}\cup \{\phi_{A}\},{\overset{\sim }{P}}_{B}^{\left(l\right)}=P_{B}^{\left(l\right)}\cup \{\phi_{B}\}$$

For any real point pair $\left(p_{i}^{A},q_{j}^{B}\right)$, its hybrid matching cost is defined as:

(19) $$c_{ij}^{\left(l\right)}=\lambda_{1}D_{\mathrm{desc}}(p_{i}^{A},q_{j}^{B})+\lambda_{2}D_{\mathrm{topo}}(p_{i}^{A},q_{j}^{B})$$

In the above expression, $\lambda_{1}$ and $\lambda_{2}$ are weighting parameters. For matching terms between a real point and a virtual node, a fixed unmatched cost $c_{\phi }$ is uniformly assigned, namely:

(20) $$c_{i\phi }^{\left(l\right)}=c_{\phi j}^{\left(l\right)}=c_{\phi }$$

The transport cost between virtual nodes is set to zero. Since the hybrid costs between real nodes have been normalized to the interval [0, 1], $c_{\phi }$ is on the same scale as the real-node costs. When the matching costs between a given real node and all real candidate nodes are relatively high, transporting its mass to a virtual node yields a lower objective value, thereby allowing the node to be identified as unmatched.

Assume that the source and target images at the current level contain $N_{s}$ and $N_{t}$ real nodes, respectively. Let ρ∈[0, 1) denote the maximum proportion of mass allowed to be assigned to virtual nodes. The augmented marginal distributions are then defined as follows:

(21) $$\overset{\sim }{\mu }={\left[\frac{1-\rho }{N_{s}},\ldots ,\frac{1-\rho }{N_{s}},\rho \right]}^{T},\overset{\sim }{\nu }={\left[\frac{1-\rho }{N_{t}},\ldots ,\frac{1-\rho }{N_{t}},\rho \right]}^{T}$$

The above construction satisfies $\underset{i}{\sum }{\overset{\sim }{\mu }}_{i}=\underset{j}{\sum }{\overset{\sim }{\nu }}_{j}=1$. Therefore, the balanced optimal transport problem in Equation (5) satisfies the feasibility condition that the total masses of the two marginal distributions are equal.

The virtual-node mass *ρ* specifies an upper bound on the unmatched mass, rather than enforcing a fixed proportion of real nodes to be classified as unmatched. Any virtual mass that is not utilized for transport involving real nodes can be transferred through the zero-cost connection between the two virtual nodes. Consequently, the proposed model can adaptively determine the actual amount of unmatched mass according to the matching costs while preserving the mass-conservation constraint of balanced optimal transport.

Through the above augmented design, some feature points can be naturally matched to virtual nodes without being forcibly assigned to incorrect correspondences, thereby improving the adaptability of the optimal transport model to dynamic outliers, missing correspondences, and spurious overlap regions.

## 4. Geometric Estimation and Map Fusion

### 4.1. RANSAC Rigid-Body Transformation Estimation

From Section 2.4, the high-confidence match set at the finest level, $S_{in}$, is obtained. Within this set, matches involving virtual nodes are treated as unmatched and are discarded, and only candidate correspondences between real feature points with high bidirectional support are retained. This match set, to a certain extent, attenuates the one-to-many matching problem caused by repetitive structures, while also reducing the impact of dynamic obstacles, partial occlusions, and temporal sampling inconsistencies on the subsequent pose estimation, thereby providing relatively reliable input for the geometric consistency verification.

Building on this, this paper employs RANSAC to perform robust estimation on the candidate matching point pairs, so as to determine the two-dimensional rigid-body transformation between the two local maps. The detailed algorithm flow is shown in Figure 5.

Assume that the transformation from the source local map to the target local map is expressed as:

(22) $$T=\left[\begin{array}{cc} R & t\\ 0^{⊤} & 1 \end{array}\right]\in SE(2)$$

In this expression, *R* is the two-dimensional rotation matrix, and *t* is the two-dimensional translation vector. RANSAC generates transformation hypotheses by randomly sampling minimal matching subsets, counts inliers based on geometric residuals, and ultimately selects the model with the highest support as the estimated relative pose between the local maps.

Compared with directly using all candidate matches for least-squares estimation, RANSAC is more robust in the presence of a small number of false matches and dynamic outliers. For the repetitive unit boundaries, similar pallet stacks, and local spurious correspondences caused by forklift and personnel movements commonly found in large-scale packaging and printing workshops, this method can effectively suppress the interference of abnormal matches on the pose estimation result. After obtaining the rigid-body transformation, the source local map can be transformed into the coordinate system of the target map, thereby providing a unified spatial reference for the subsequent grid probability fusion.

### 4.2. Log-Odds Probability Fusion

After completing the alignment of the local maps, this paper adopts the log-odds form for probabilistic fusion. Compared with directly applying overwrite updating or simple averaging to binary occupancy maps, the log-odds representation can accumulate information from multiple observations more naturally, and is thus more suitable for multi-robot local map fusion tasks.

Let the log-odds values at grid cell *g* of the aligned source map and the target map be $L_{A}(g)$ and $L_{B}(g)$, respectively, and let the prior term be $L_{0}$. Assuming that the two observations are independent and equally reliable, an equal-weight fusion strategy is adopted, and the fused log-odds value is given by:

(23) $$L_{\mathrm{fuse}}(g)=L_{A}(g)+L_{B}(g)-L_{0}$$

This formulation is equivalent to the standard Bayesian update under independent observations, which is concise in form and has a clear probabilistic interpretation. The fused occupancy probability can then be expressed as:

(24) $$p_{\mathrm{fuse}}(g)=\frac{1}{1+exp(-L_{\mathrm{fuse}}(g))}$$

The current implementation adopts an equal-weight log-odds fusion model under the assumption of independent and equally reliable observations. For conflicting occupancy observations, opposite-signed log-odds values partially compensate for each other, which can reduce the influence of transient inconsistencies. However, the model does not explicitly account for observation reliability, temporal decay, or stale occupancy information. Therefore, dynamic-disturbance robustness in this study is mainly provided by the preceding correspondence filtering, virtual-node matching, and RANSAC estimation stages. Reliability-weighted or time-aware fusion will be considered in future work.

## 5. Experimental Validation

### 5.1. Experimental Setup and Datasets

To validate the effectiveness of the proposed method in scenarios of intelligent factories with densely arranged electromechanical equipment, a simulated packaging and printing workshop environment was constructed. This environment was built with reference to the layout of a real workshop and includes a printing unit area, a main corridor area, a finished product stacking area, a die-cutting area, a loading and unloading area, and a large-scale cross-regional scene. All programs in this study were executed on a laptop equipped with an AMD Ryzen 7 4800H CPU and 32 GB of RAM. The reported runtime was measured on a laptop platform rather than on the onboard computers. Therefore, the present results characterize the computational performance under the specified hardware configuration but do not demonstrate real-time execution on the Raspberry Pi 4 (Raspberry Pi Ltd., Cambridge, UK) or Orange Pi 5 Pro (Shenzhen Xunlong Software Co., Ltd., Shenzhen, China). Further platform-specific optimization and embedded evaluation are required for onboard deployment.

Data collection was carried out cooperatively by two heterogeneous mobile robots, and the experimental equipment is shown in Figure 6.

The hardware platform configurations are as follows:

(1) Robot A adopts a Mecanum wheel omnidirectional mobile structure. Its onboard computing unit is a Raspberry Pi 4, also running Ubuntu 22.04 and ROS 2. The chassis control is implemented by an STM32 microcontroller (NUCLEO-F303RE development board, STMicroelectronics, Geneva, Switzerland) to achieve coordinated driving of the omnidirectional wheels.

(2) Robot B adopts an Ackermann steering structure. Its onboard computing unit is an Orange Pi 5 Pro running the Ubuntu 22.04 operating system and the Robot Operating System 2 (ROS 2) framework. The chassis control is based on an STM32 microcontroller main controller, which is responsible for closed-loop wheel speed control and steering actuation. It is equipped with a 2D LiDAR for environmental perception and occupancy grid map construction.

The two mobile robots independently collected local occupancy grid maps in the simulated packaging and printing workshop environment at different times and along different trajectories, forming the self-constructed simulated workshop dataset. Before collecting local maps, a single mobile robot was employed to traverse the entire simulated workshop environment, and a complete global occupancy grid map was generated using the Cartographer LiDAR-based SLAM framework. This global map was used as the reference map for establishing the ground truth coordinate system.

The dataset contains 10 independent local map pairs generated from different robot trajectories. Each local map pair has an average overlapping ratio of approximately 50%. The simulated workshop scene covers an area of 18 m × 16 m, and all occupancy grid maps are generated with a resolution of 0.1 m/cell, corresponding to a grid size of 180 × 160 cells. Robot A is equipped with an RPLIDAR A1 two-dimensional LiDAR sensor, while Robot B is equipped with an RPLIDAR C1 two-dimensional LiDAR sensor for environmental perception and map construction. The different sensing characteristics of the two LiDAR sensors provide heterogeneous observation conditions for multi-robot map registration.

To evaluate the stability and repeatability of the proposed method, each independent map pair was tested 50 times under the same experimental configuration, resulting in 500 repeated evaluations in total. The reference transformation between two local maps was obtained from their poses in the global coordinate system. Specifically, the poses of the two local maps relative to the reference global map were first determined, and the ground-truth transformation between the source and target maps was calculated according to their relative pose relationship. The rotation and translation errors reported in this paper are calculated by comparing the estimated transformation with the corresponding ground-truth transformation.

The local maps generated by different robots under varying trajectories and acquisition times contain realistic differences caused by sensor viewpoints, observation coverage, local occlusions, temporarily stored objects, and dynamic environmental changes. These variations provide a challenging evaluation scenario for multi-robot occupancy-grid map registration and fusion in large-scale simulated industrial environments.

### 5.2. Comparative Methods and Evaluation Metrics

For comparison, ORB-RANSAC, OT-Baseline, Correlative Registration, and the proposed method were evaluated. ORB-RANSAC establishes feature correspondences using ORB descriptors and estimates the two-dimensional rigid-body transformation using RANSAC. OT-Baseline was implemented in this study as a single-resolution optimal-transport baseline. It extracts ORB features at the original resolution, constructs an augmented matching model using local neighborhood graphs and virtual nodes, solves feature correspondences through entropy-regularized optimal transport, and finally estimates the rigid-body transformation using RANSAC.

Correlative Registration was introduced as an occupancy-grid-based baseline. It estimates the relative transformation by searching the translation–rotation space and maximizing the correlation between the two occupancy grid maps. The transformation with the highest correlation score is selected as the registration result.

The proposed method further incorporates multi-resolution cascading, local topological signatures, dynamically regularized Sinkhorn iterations, cross-layer prior transfer, high-confidence correspondence filtering, and log-odds-based probabilistic fusion.

To conduct a multi-dimensional evaluation covering registration accuracy, computational efficiency, registration reliability, and fusion performance, the following evaluation metrics are adopted in this study:

(1) For evaluating computational efficiency, the elapsed time from reading the local maps to outputting the fused map is adopted. This metric covers the complete pipeline of feature extraction, registration estimation, and map fusion.

(2) For evaluating map registration accuracy, a joint description using rotation error and translation error is adopted. The rotation error and translation error together constitute a complete accuracy measure for the rigid-body transformation.

(3) For evaluating the overall performance and engineering reliability of the algorithm, the proportion of samples satisfying a rotation error < 1.0°and a translation error < 0.30 m is adopted. The success rate can condense discrete judgments from individual experiments into an overall probability, preventing the mean value from masking extreme failure cases.

(4) For evaluating the quality of the final fused map, the Intersection over Union (IoU) of occupied grids between the fused map and the reference global map is adopted. IoU can simultaneously measure three types of errors—correct occupancy, missed occupancy, and false occupancy—and can thus integrally reflect the completeness, accuracy, and consistency of the fused map.

The key parameters used in this study and their corresponding settings are summarized in Table 1.

The main hyperparameters were selected according to the design requirements of the proposed framework and were fixed for all evaluations. The six ORB settings in Table 1 correspond to the maximum number of features, scale factor, number of internal pyramid levels, edge threshold, patch size, and FAST threshold, respectively. The Sinkhorn iteration terminates when the maximum change in the dual variables between consecutive iterations falls below 1 × 10^−6^, or when the maximum number of 250 iterations is reached. The RANSAC inlier threshold is 3 grid cells, corresponding to 0.30 m at the map resolution of 0.1 m/cell.

### 5.3. Comparative Experiments and Low-Overlap Robustness Experiments

To evaluate the overall performance of the proposed method, a total of 500 repeated evaluations were conducted on 10 independent local-map pairs from the packaging and printing workshop dataset. ORB-RANSAC, OT-Baseline, Correlative Registration, and the proposed method were evaluated under the same experimental conditions. Figure 7 shows a representative pair of local occupancy grid maps with overlapping regions from the self-constructed packaging and printing workshop dataset. The two maps were acquired by mobile robots along different trajectories and exhibit partial structural overlap, while also reflecting variations caused by differences in sensing coverage, motion paths, and environmental conditions.

Figure 8 compares the feature-matching results obtained by the three methods on the self-constructed packaging and printing workshop dataset.

Figure 9 presents the occupancy grid map stitching results obtained using the three methods on the self-constructed packaging and printing workshop dataset.

Table 2 summarizes the rotation error, translation error, total runtime, registration success rate, and occupancy-grid fusion IoU obtained by ORB-RANSAC, OT-Baseline, Correlative Registration, and the proposed method on the self-constructed simulated packaging and printing workshop dataset. The reported results are expressed as mean ± standard deviation across the independent map pairs.

As shown in Table 2, the proposed method achieves the lowest rotation and translation errors among the four comparison methods. The mean rotation error is 0.37 ± 0.11°, corresponding to reductions of 58.0%, 42.2%, and 48.6% compared with ORB-RANSAC (0.88 ± 0.26°), OT-Baseline (0.64 ± 0.19°), and Correlative Registration (0.72 ± 0.23°), respectively. Similarly, the mean translation error of the proposed method is 0.09 ± 0.03 m, which is 59.1%, 35.7%, and 47.1% lower than those of ORB-RANSAC (0.22 ± 0.07 m), OT-Baseline (0.14 ± 0.05 m), and Correlative Registration (0.17 ± 0.06 m), respectively. The relatively small standard deviations further indicate that the proposed method maintains stable registration accuracy across different map pairs. These improvements can be attributed to the combined effects of multi-resolution cascading, local topological constraints, virtual-node-based partial matching, and cross-layer prior guidance, which reduce matching ambiguities and suppress unreliable correspondences.

In terms of computational efficiency, the proposed method requires an average runtime of 0.78 ± 0.07 s, which is lower than those of OT-Baseline (0.92 ± 0.09 s) and Correlative Registration (1.12 ± 0.14 s), although it remains higher than that of ORB-RANSAC (0.43 ± 0.05 s). The reduced runtime relative to the single-resolution OT and correlation-based methods indicates that coarse-to-fine candidate reduction and cross-layer prior guidance can partially compensate for the additional computational cost introduced by the multi-stage matching framework. Therefore, the proposed method provides a reasonable balance between registration accuracy and computational efficiency.

Regarding registration reliability, the proposed method achieves a success rate of 98.4 ± 2.2%, compared with 85.3 ± 6.8% for ORB-RANSAC, 93.7 ± 4.5% for OT-Baseline, and 90.6 ± 5.7% for Correlative Registration. The proposed method also obtains the highest grid-fusion IoU of 0.96 ± 0.02, whereas ORB-RANSAC, OT-Baseline, and Correlative Registration achieve 0.89 ± 0.04, 0.92 ± 0.03, and 0.91 ± 0.03, respectively. These results indicate that the improved relative-pose estimation contributes to better spatial consistency of the fused occupancy grid maps. Overall, the experimental results demonstrate that the proposed method provides improved registration accuracy, stability, and fusion consistency while maintaining moderate computational cost in the simulated packaging and printing workshop environment.

To quantitatively evaluate the independent contribution of each core component in the proposed method, ablation experiments were conducted on the multi-resolution cascading strategy, cross-layer prior, warm-start initialization, local topological signatures, and virtual-node-based partial matching. In each experiment, only one component was removed from the complete method, while all other settings were kept unchanged. The results are presented in Table 3.

The ablation study demonstrates the distinct contributions of the main components of the proposed method. The multi-resolution cascading mechanism reduces the matching search space and improves computational efficiency while preserving registration accuracy. The cross-layer prior improves the reliability of fine-level matching by transferring coarse-level structural information to constrain the optimization process, thereby reducing ambiguity in repetitive environments. In contrast, the warm-start mechanism primarily improves computational efficiency by initializing the fine-level dual variables with the converged solution from the preceding coarse level, thereby reducing computational time with limited influence on the final registration accuracy.

The local topological signatures enhance feature discriminability by incorporating neighborhood structural information, which improves correspondence reliability in scenes containing repetitive geometric structures. The virtual-node-based partial matching mechanism accommodates missing correspondences and outliers, preventing unreliable features from being forcibly assigned to incorrect matches and improving robustness under partial occlusion and dynamic disturbances. Overall, these components play complementary roles in registration accuracy, convergence efficiency, and robustness, supporting the effectiveness and rationality of the proposed multi-resolution cascaded optimal-transport framework.

To further evaluate the robustness of the proposed method to dynamic environmental changes, controlled occupancy perturbations were introduced into the original map pairs. For each map pair, one map was kept unchanged, while temporary occupied regions were inserted into the valid overlapping free-space area of the other map to simulate local environmental changes caused by temporarily stored objects or mobile equipment. Three perturbation levels were considered by varying the number of inserted obstacle regions, and the corresponding map-change ratio was calculated as the proportion of modified grid cells within the valid overlapping region. OT-Baseline and the proposed method were evaluated under identical parameter settings, and the results are presented in Table 4.

As shown in Table 4, the performance of both methods decreases as the dynamic perturbation level increases. However, the proposed method exhibits smaller increases in rotation and translation errors and maintains a higher success rate than OT-Baseline. This indicates that the proposed method is less sensitive to local occupancy changes and provides more stable registration under dynamic map perturbations, mainly due to the local topological constraints, virtual-node-based partial matching, and high-confidence correspondence filtering.

To systematically evaluate the effect of overlap ratio, submap pairs with 20%, 40%, and 60% overlap were generated by cropping the global map of the packaging and printing workshop, and a comparison was conducted between OT-Baseline and the proposed method. The results are shown in Table 5.

The experimental results demonstrate that, in the multi-robot grid map fusion task for the packaging and printing workshop, the proposed method outperforms ORB-RANSAC and OT-Baseline in registration accuracy, success rate, and fusion quality. Under low-overlap conditions, the proposed method still maintains a high success rate, validating the robustness of multi-resolution cascading and partial matching with virtual nodes to sparse observations. Moreover, the proposed method achieves higher registration accuracy with fewer matches, and its total runtime is faster than that of OT-Baseline.

## 6. Conclusions

### 6.1. Discussion

The experimental results demonstrate that the proposed method achieves favorable map registration and fusion performance on the self-constructed packaging and printing workshop dataset. Compared with ORB and OT-Baseline, the proposed method reduces the rotation and translation errors to 0.37° and 0.09 m, respectively, while achieving a registration success rate of 98.4% and a fusion IoU of 0.96. These results indicate that the proposed multi-resolution cascading strategy, cross-layer prior transfer mechanism, and local topological constraints can improve matching stability in environments characterized by repetitive structures. In addition, the proposed method requires an average runtime of 0.78 s, which is 15.2% shorter than that of OT-Baseline at 0.92 s. This suggests that candidate search-space reduction at coarse resolutions can partially compensate for the additional computational cost introduced by multi-stage matching and optimal transport optimization.

The low-overlap experiments further show that the registration errors of both optimal-transport-based methods increase as the map overlap decreases, whereas the proposed method maintains more stable performance. At an overlap ratio of 20%, the proposed method achieves a rotation error of 0.54°, a translation error of 0.21 m, and a registration success rate of 93.3%, outperforming OT-Baseline. These results suggest that multi-resolution cascading, local structural constraints, and virtual-node-based partial matching help preserve stable geometric estimation when the number of valid correspondences is reduced. Overall, the proposed method achieves a favorable balance among registration accuracy, fusion quality, and computational efficiency, demonstrating its potential for structured industrial environments such as large-scale packaging and printing workshops.

### 6.2. Summary of Conclusions

This study addresses the challenges of large map scales, pronounced structural repetition, limited local overlap, and frequent dynamic disturbances in large-scale packaging and printing workshops by proposing a multi-robot occupancy grid map fusion method based on multi-resolution cascading and optimal transport. The proposed method constructs a coarse-to-fine multi-resolution matching representation to reduce the candidate search space and employs cross-layer prior transfer to guide fine-resolution matching. ORB-based appearance descriptors and local topological signatures are jointly incorporated to formulate a hybrid matching cost, while virtual nodes are introduced to handle missing correspondences and anomalous matches. Local map registration and fusion are subsequently achieved through log-domain Sinkhorn iterations, high-confidence correspondence filtering, RANSAC-based rigid-body transformation estimation, and Log-odds-based probabilistic fusion. The experimental results demonstrate the effectiveness of the proposed method in complex industrial environments.

This study still has several limitations. The current experiments were conducted primarily in a single self-constructed packaging and printing workshop environment, while the low-overlap experiments were generated by cropping the global map to obtain local map pairs with different overlap ratios. Therefore, the generalization capability of the proposed method across different workshop layouts, robotic platforms, and more complex dynamic environments requires further validation.

## Figures and Tables

**Figure 1:**
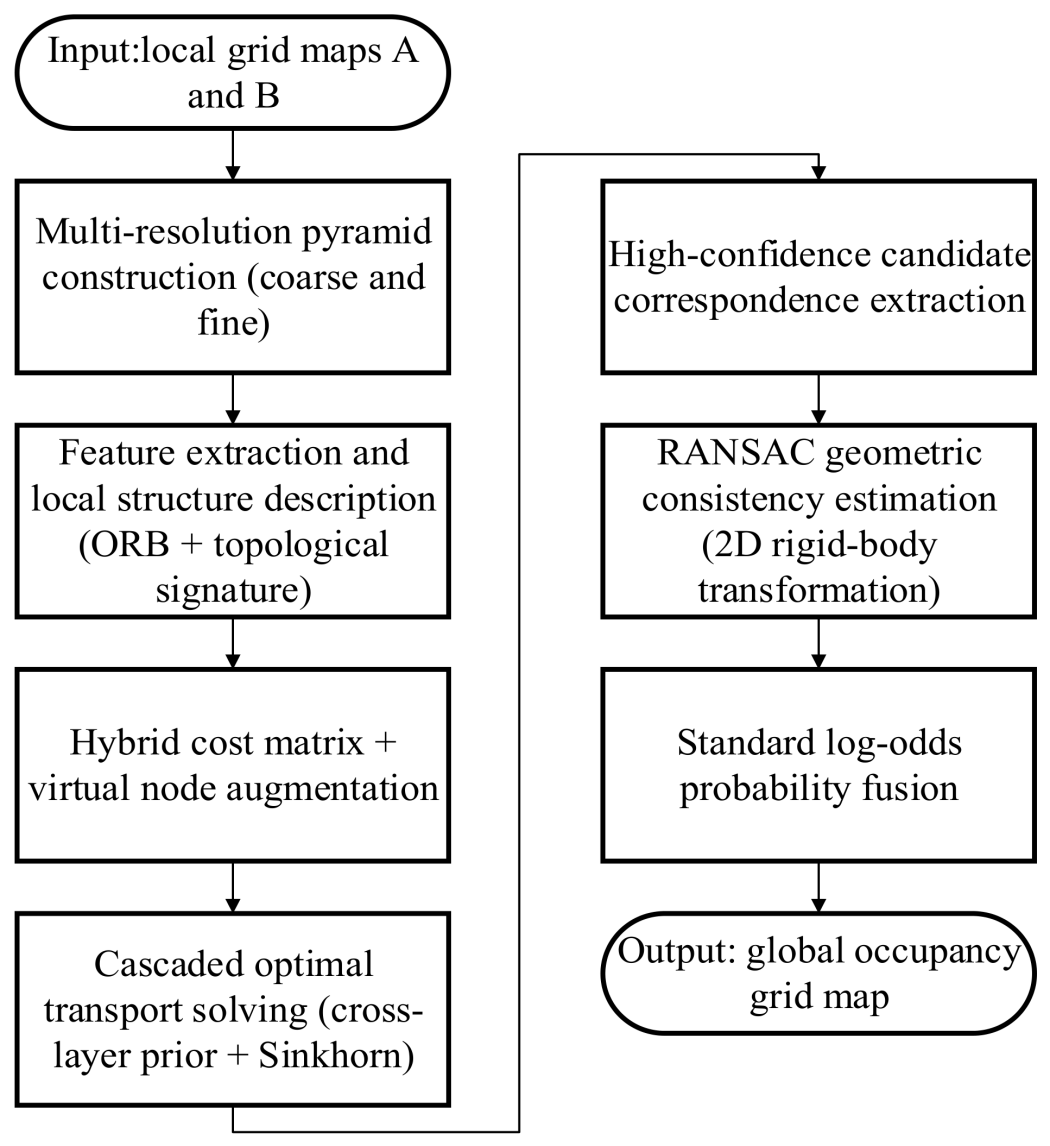
Block diagram of the multi-resolution cascaded optimal transport fusion algorithm.

**Figure 2:**
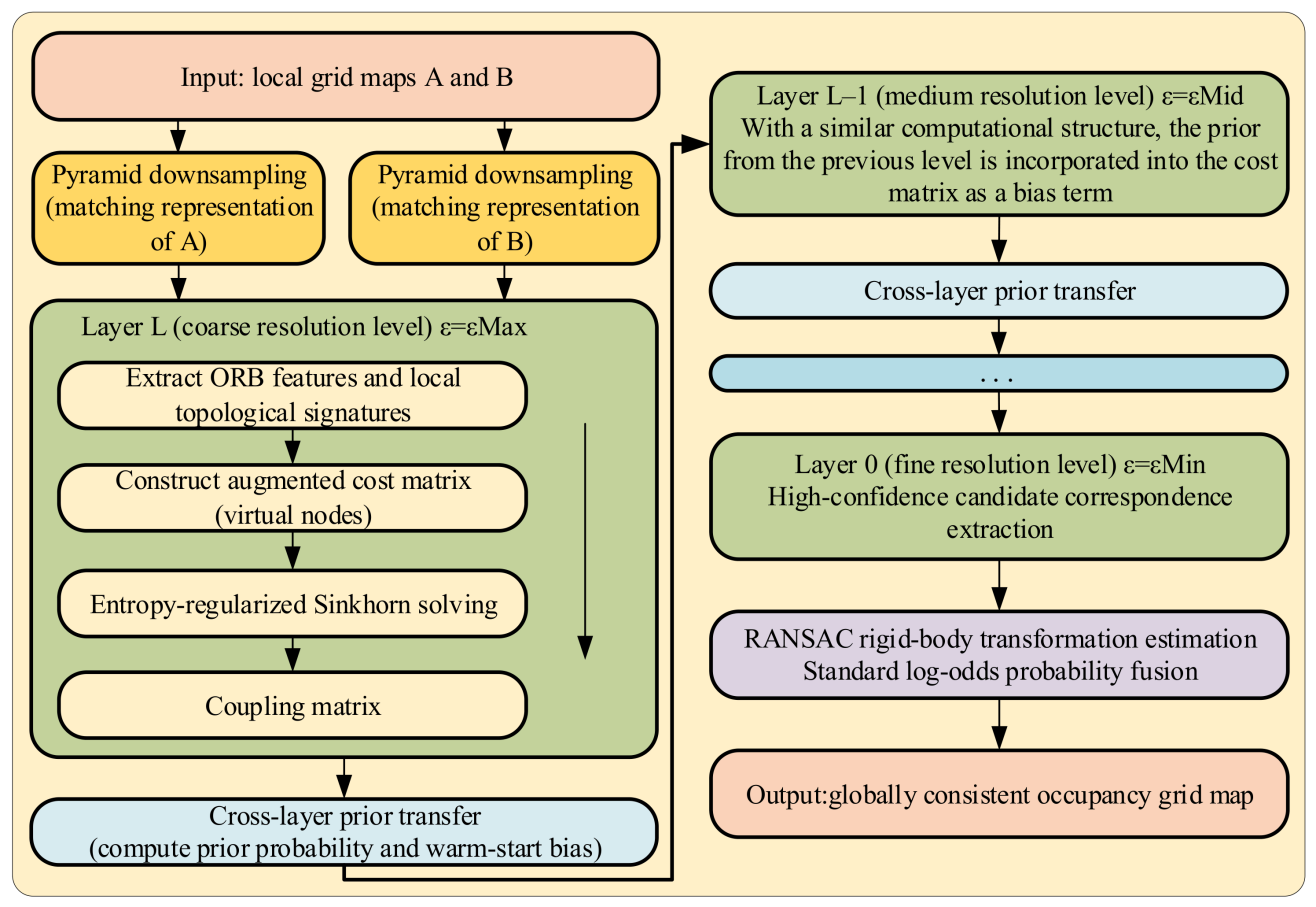
Multi-resolution cascaded pyramid model.

**Figure 3:**
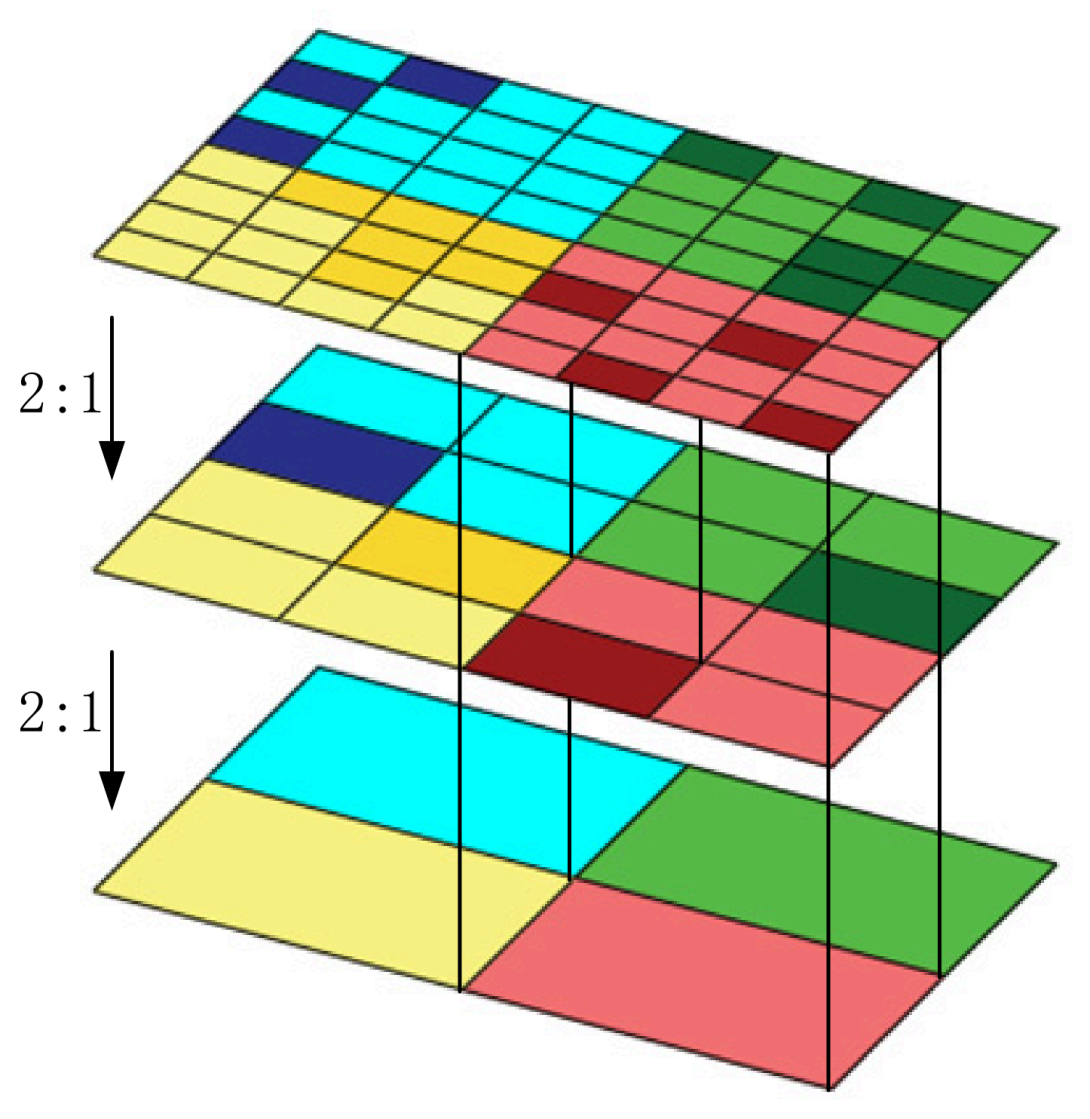
Schematic diagram of Gaussian downsampling of the local grid map.

**Figure 4:**
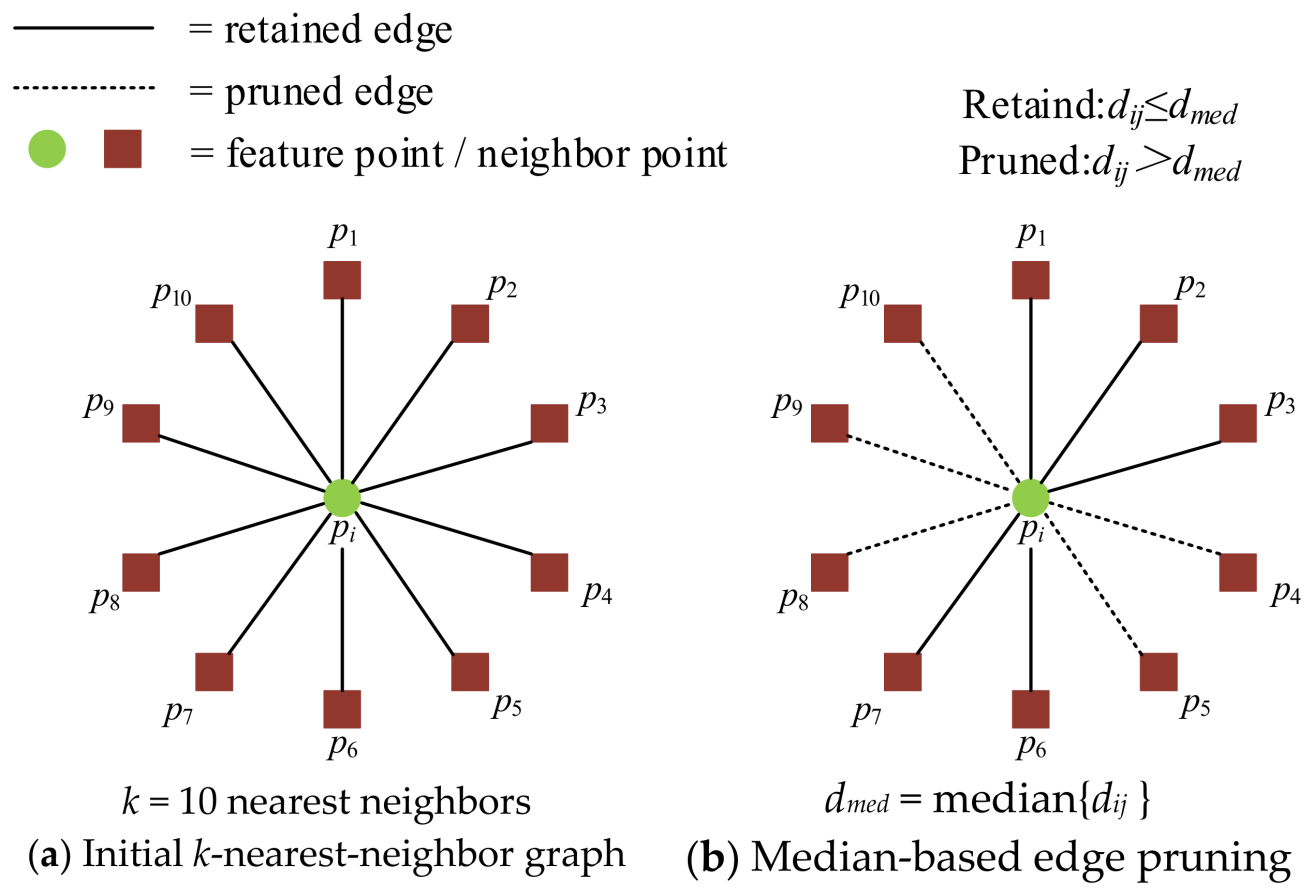
Median-based k-nearest-neighbor pruning.

**Figure 5:**
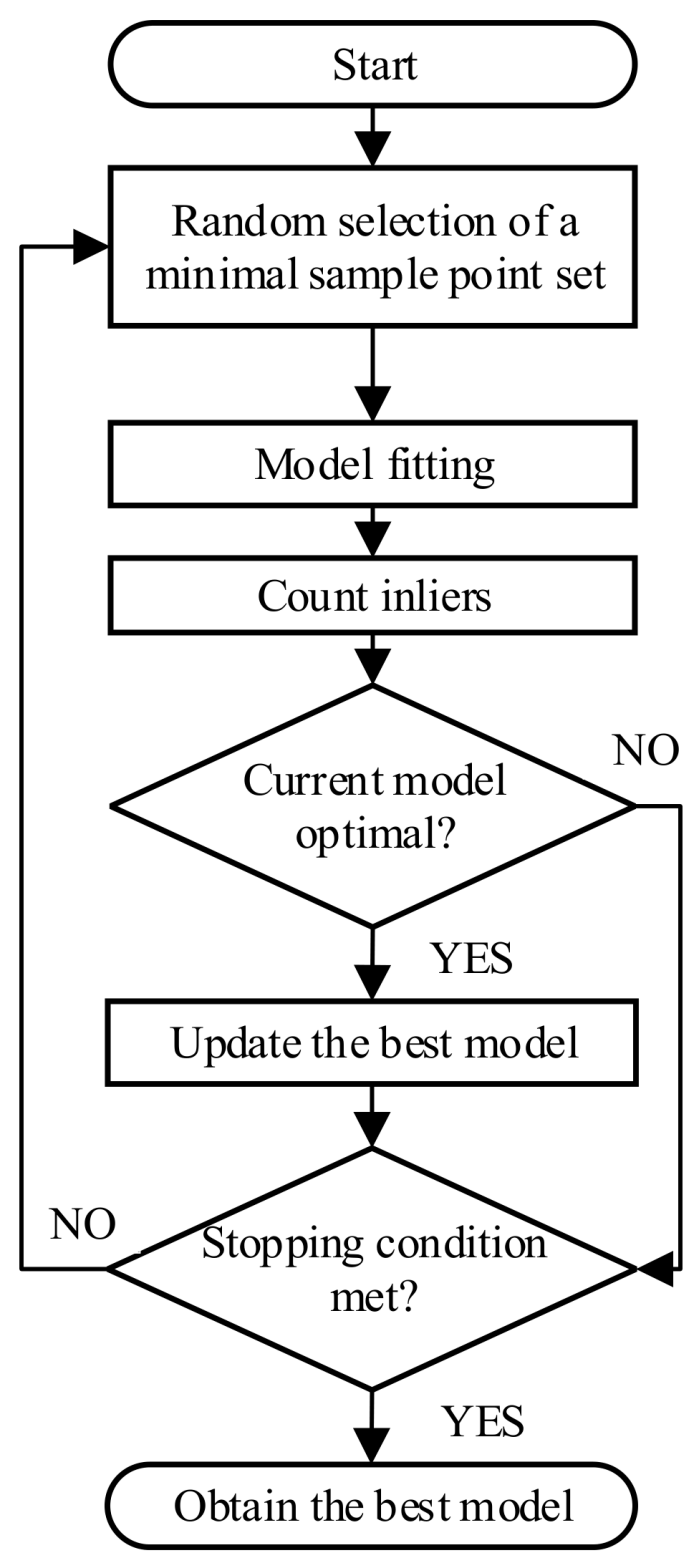
Flowchart of the RANSAC algorithm.

**Figure 6:**
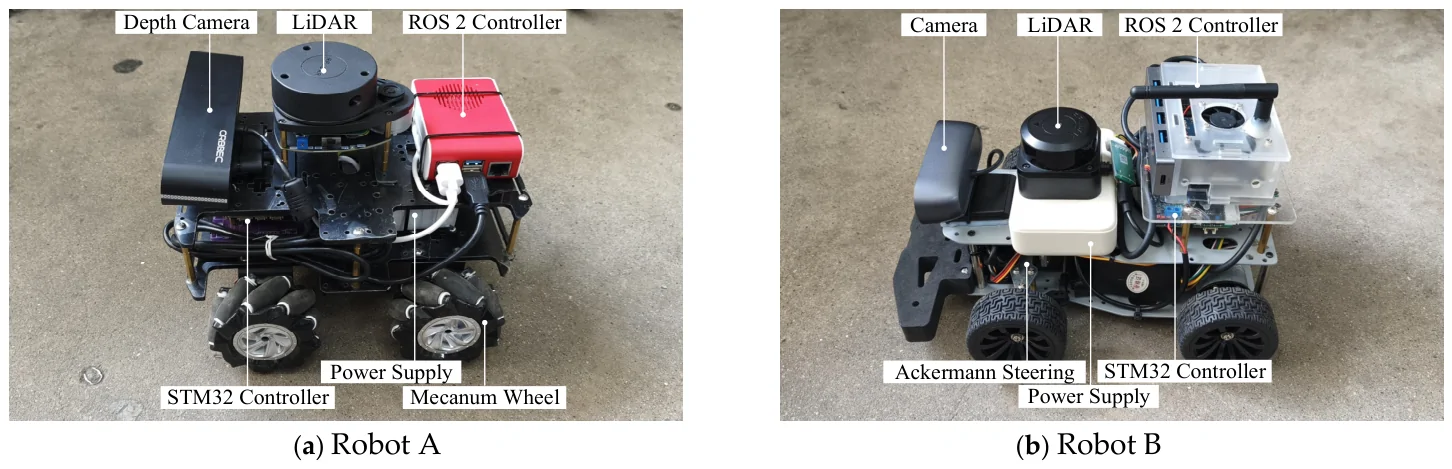
Two experimental robots equipped with LiDAR.

**Figure 7:**
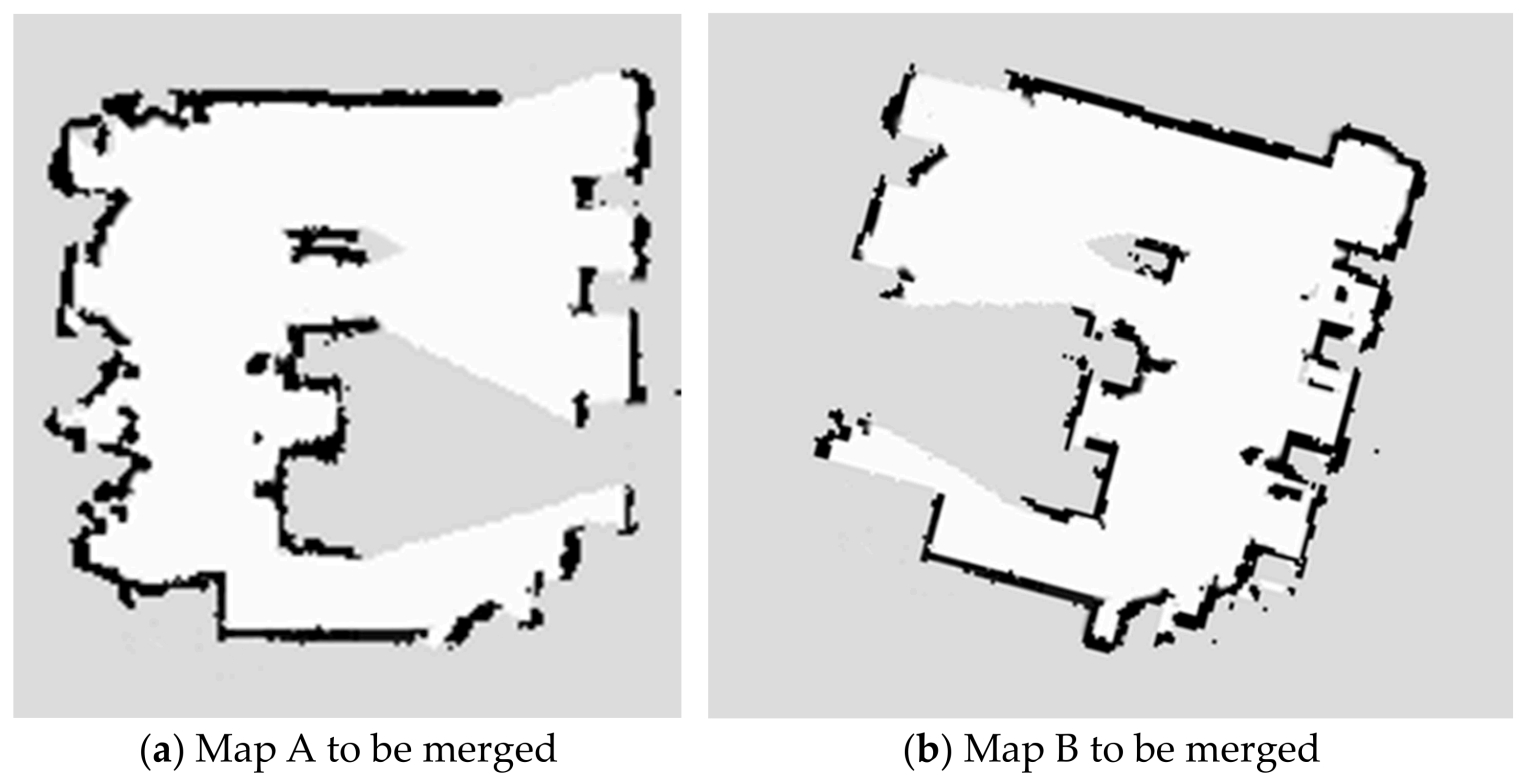
Grid maps of the simulated packaging and printing workshop to be stitched.

**Figure 8:**
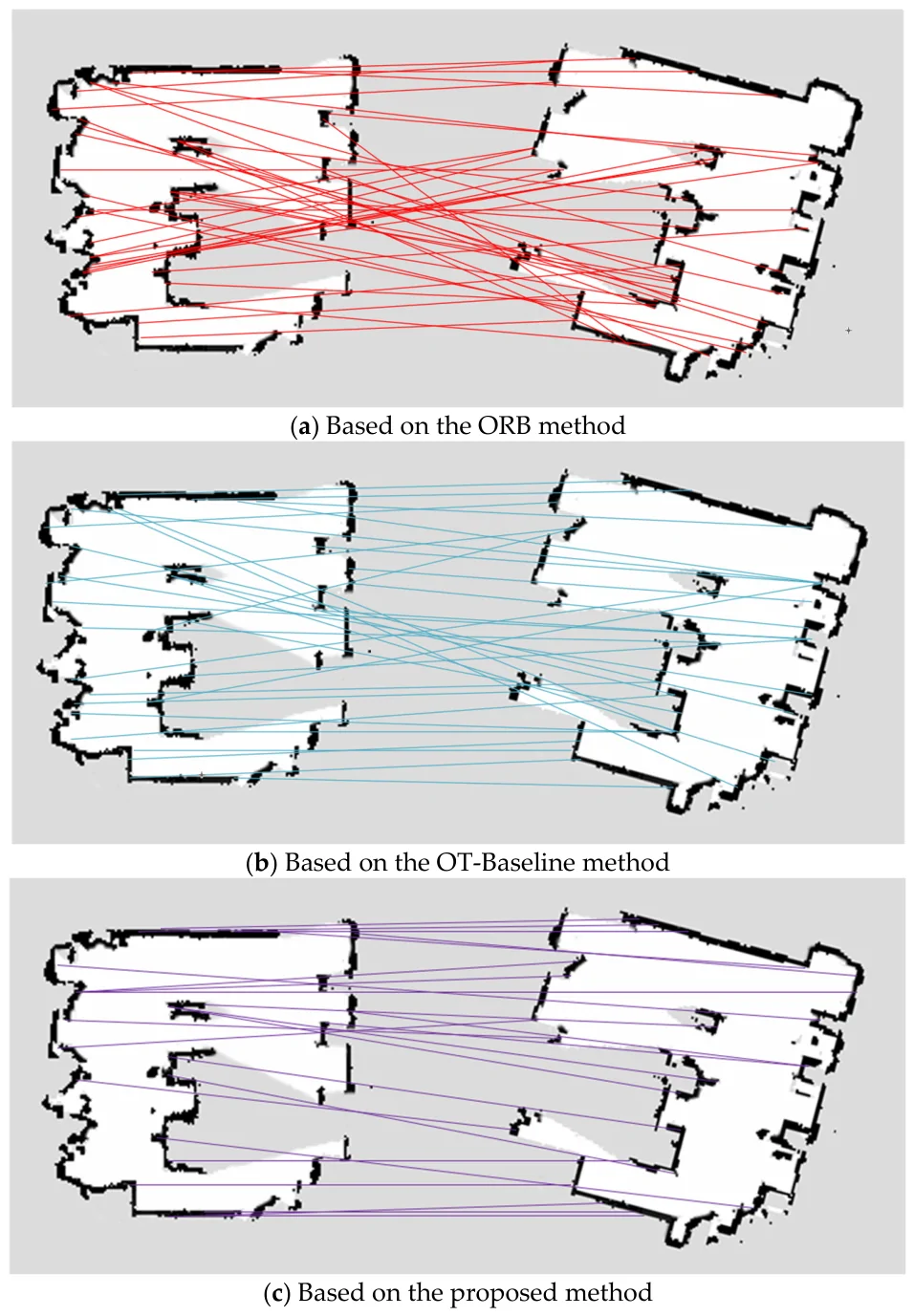
Feature matching results of the packaging and printing workshop.

**Figure 9:**
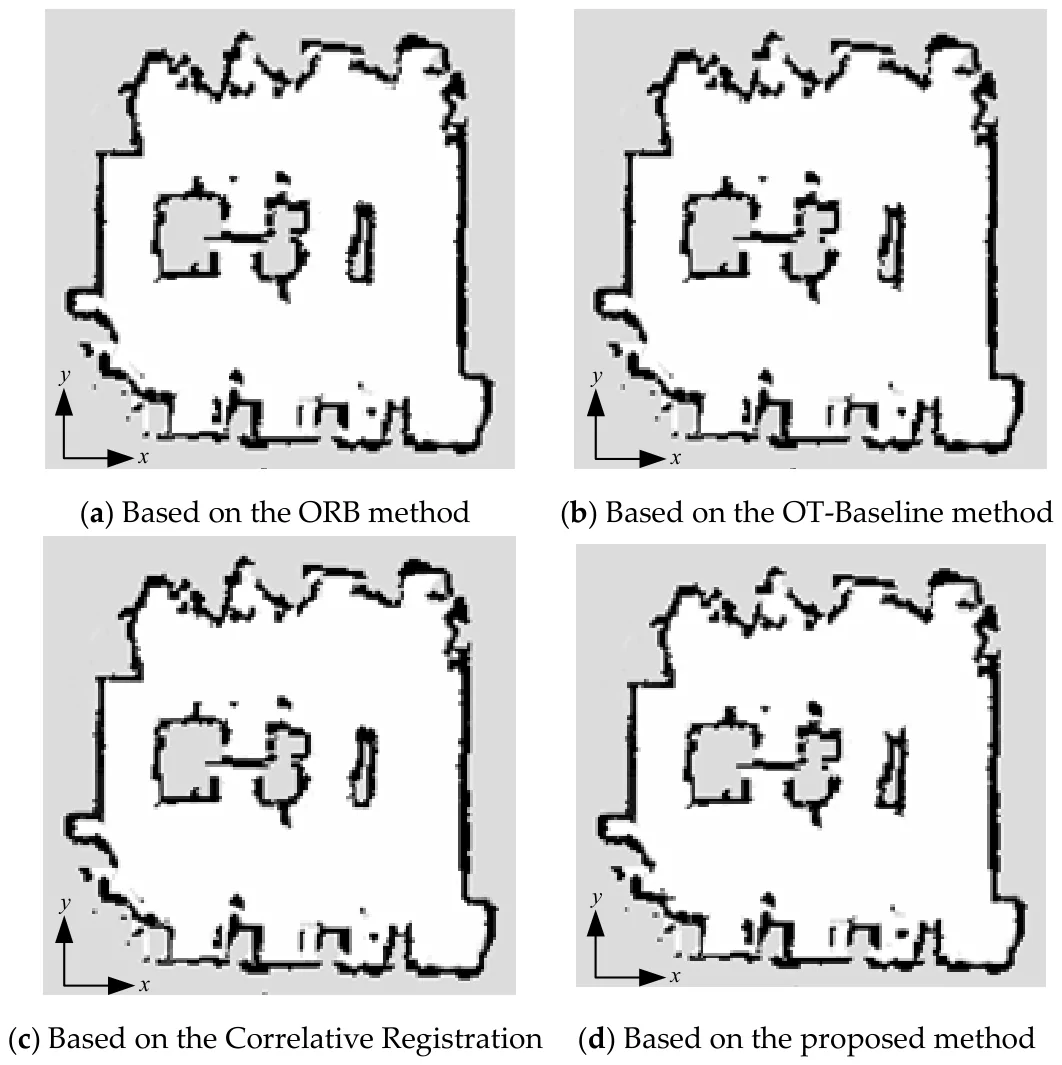
Grid map stitching result of the packaging and printing workshop.

**Table 1:** Summary of key parameter settings.

Parameter	Symbol	Setting
Number of pyramid levels	*L*	3
Downsampling ratio		2:1
*k*-nearest-neighbor parameter	*k*	10
Hybrid cost weights	$\lambda_{1},\lambda_{2},$	0.6, 0.4
Maximum Unmatched Mass Ratio	*ρ*	0.2
Unmatched cost	$c_{\phi }$	0.7
Regularization coefficients at different levels	$\varepsilon_{0},\varepsilon_{1},\varepsilon_{2}$	0.01, 0.05, 0.1
RANSAC inlier threshold	$d_{in}$	3 grid cells
Confidence threshold	*η*	0.05
Cross-layer prior weights	$\beta_{0},\beta_{1},\beta_{2}$	0.2, 0.2, 0
ORB extraction settings		1500/1.2/8/10/31/10
Sinkhorn convergence tolerance	τ	1 × 10^−6^
Maximum Sinkhorn iterations	$N_{S}$	250
RANSAC confidence		0.99
Maximum RANSAC iterations	$N_{R}$	3000

**Table 2:** Comparative experimental results based on the packaging and printing workshop dataset.

Method	Rotation Error (°)	Translation Error (m)	Runtime (s)	Success Rate (%)	Grid Fusion IoU
ORB	0.88 ± 0.26	0.22 ± 0.07	0.43 ± 0.05	85.3 ± 6.8	0.89 ± 0.04
OT-Baseline	0.64 ± 0.19	0.14 ± 0.05	0.92 ± 0.09	93.7 ± 4.5	0.92 ± 0.03
Correlative Registration	0.72 ± 0.23	0.17 ± 0.06	1.12 ± 0.14	90.6 ± 5.7	0.91 ± 0.03
Proposed method	0.37 ± 0.11	0.09 ± 0.03	0.78 ± 0.07	98.4 ± 2.2	0.96 ± 0.02

**Table 3:** Ablation study results of each module of the proposed method.

Method	Rotation Error (°)	Translation Error (m)	Success Rate (%)	Runtime (s)
Full method	0.37 ± 0.11	0.09 ± 0.03	98.4 ± 2.2	0.78 ± 0.07
Without multi-resolution cascading	0.62 ± 0.18	0.15 ± 0.05	92.6 ± 4.8	0.98 ± 0.10
Without cross-layer prior	0.42 ± 0.14	0.12 ± 0.04	95.4 ± 3.4	0.86 ± 0.08
Without warm start	0.40 ± 0.12	0.10 ± 0.03	97.5 ± 2.6	0.91 ± 0.07
Without local topological signatures	0.58 ± 0.17	0.14 ± 0.05	91.7 ± 5.2	0.82 ± 0.08
Without virtual-node-based partial matching	0.78 ± 0.24	0.21 ± 0.07	89.5 ± 6.1	0.89 ± 0.09

**Table 4:** Registration performance under different levels of dynamic map perturbation.

Dynamic Level	Temporary Obstacles	Change Ratio (%)	Method	Rotation Error (°)	Translation Error (m)	Success Rate (%)
Original	0	0	OT-Baseline	0.64 ± 0.19	0.14 ± 0.05	93.7 ± 4.5
			Proposed method	0.37 ± 0.11	0.09 ± 0.03	98.4 ± 2.2
Low	3	4.8	OT-Baseline	0.71 ± 0.22	0.17 ± 0.06	91.2 ± 5.1
			Proposed method	0.40 ± 0.12	0.10 ± 0.03	97.2 ± 2.6
Medium	6	9.5	OT-Baseline	0.84 ± 0.27	0.21 ± 0.07	86.8 ± 6.4
			Proposed method	0.46 ± 0.14	0.12 ± 0.04	95.1 ± 3.3
High	9	14.7	OT-Baseline	1.03 ± 0.35	0.29 ± 0.10	78.5 ± 8.0
			Proposed method	0.56 ± 0.18	0.16 ± 0.05	91.4 ± 4.9

**Table 5:** Registration performance under different overlap rates in the packaging and printing workshop.

Overlap Ratio	Method	Rotation Error (°)	Translation Error (m)	Success Rate (%)
60%	OT-Baseline	0.42 ± 0.12	0.15 ± 0.04	97.6 ± 2.8
	Proposed method	0.32 ± 0.09	0.12 ± 0.03	98.1 ± 2.1
40%	OT-Baseline	0.58 ± 0.17	0.21 ± 0.06	93.3 ± 4.4
	Proposed method	0.41 ± 0.12	0.17 ± 0.05	96.7 ± 3.1
20%	OT-Baseline	0.89 ± 0.28	0.32 ± 0.09	83.3 ± 5.2
	Proposed method	0.54 ± 0.16	0.21 ± 0.06	93.3 ± 4.8

## Data Availability

The original contributions presented in this study are included in the article. Further inquiries can be directed to the corresponding author.
